# B cells induced by *Schistosoma japonicum* infection display diverse regulatory phenotypes and modulate CD4^+^ T cell response

**DOI:** 10.1186/s13071-020-04015-3

**Published:** 2020-03-20

**Authors:** Junli Xiao, Fei Guan, Li Sun, Yijie Zhang, Xiaoyan Zhang, Shengjun Lu, Wenqi Liu

**Affiliations:** Department of Parasitology, School of Basic Medicine, Tongji Medical College, Huazhong University of Science and Technology, Wuhan, China

**Keywords:** *Schistosoma japonicum*, Regulatory B cells, Th responses, PD-L1, IL-10

## Abstract

**Background:**

The increased activity of regulatory B cells (Breg) is known to be involved in immunosuppression during helminth infection, which is characterized by inducing IL-10-producing Breg cells. However, the current knowledge of B cell subsets differentiation and IL-10-independent immunoregulatory mechanisms of B cells in schistosomiasis is insufficient.

**Methods:**

BALB/c mice were percutaneously infected with cercariae for investigating the profile of B cell subsets during *Schistosoma japonicum* infection. B cells isolated from the spleen or peritoneal cavity were analyzed for the regulatory phenotype after stimulation with soluble egg antigens (SEA) *in vitro*. CD4^+^ T cells were then cocultured with B cells pretreated with or without anti-PD-L1 antibody for investigating the role of B cells from infected mice on regulating CD4^+^ T cells. Furthermore, the *in vivo* administration of anti-PD-L1 antibody was conducted to investigate the role of PD-L1 in regulating host immunity during infection.

**Results:**

The percentages of peritoneal and splenic B-1a cells, as well as marginal zone B (MZB) cells were decreased at eight and twelve weeks after infection compared to those from uninfected mice. In splenic B cells, TGF-β expression was increased at eight weeks but declined at twelve weeks of infection, and PD-L1 expression was elevated at both eight and twelve weeks of infection. In addition, SEA stimulation *in vitro* significantly promoted the expression of IL-10 in peritoneal B cells and CD5 in splenic B cells, and the SEA-stimulated splenic and peritoneal B cells preferentially expressed PD-L1 and TGF-β. The splenic B cells from infected mice were able to suppress the function of Th1 and Th2 cells *in vitro* but to expand the expression of Tfh transcription factor Bcl6, which was further enhanced by blocking PD-L1 of B cells before co-cultivation. Moreover, Th2 response and Bcl6 expression in CD4^+^ T cells were also increased *in vivo* by blocking PD-L1 after infection, although the hepatic pathology was slightly influenced.

**Conclusions:**

Our findings revealed that *S. japonicum* infection modulates the differentiation of B cell subsets that have the capability to affect the CD4^+^ T cell response. This study contributes to a better understanding of B cells immune response during schistosomiasis.
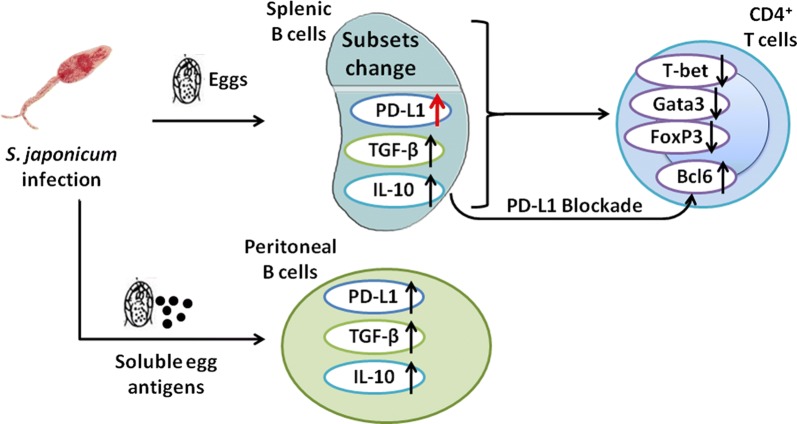

## Background

Schistosomes are pathogens that preferentially stimulate Th2 cells after the deposition of eggs in tissues [1]. Chronic schistosome infection is characterized by the downregulation of the immune system, which is widely considered to be associated with a decreased prevalence of inflammatory diseases in their hosts [2, 3]. Recent studies have revealed that apart from regulatory T cells (Treg), B cells can also be involved in this process. Most experimental studies on schistosome-induced regulatory B cells have focused on *Schistosoma mansoni* [4, 5]. However, there are remarkable differences in the development and progression of immunopathology and immune modulation between *S. mansoni* and *S. japonicum* [6].

B cells are conventionally considered to participate in immune responses by producing antibodies. Recently, the regulatory property of B cells has been reported in many inflammatory diseases [7]. The phenotypes of Breg cells have been studied, and B cell subsets with regulatory functions include transitional 2 marginal zone B precursor (T2-MZP) cells [8], MZB cells [5, 9], B10 cells and B-1a cells [10–12]. In addition to IL-10, Breg cells have been increasingly reported to play immunomodulatory role through other mechanisms. More recently, TGF-β-producing Breg cells have been reported to control the inflammation in autoimmune diabetes [13]. Besides, Breg cells characterized by elevated PD-L1 suppress autoimmune disease and anti-tumor immune response through inhibiting antibody production and T cells activation [14–17]. PD-L1 is constitutively expressed in various murine immune cells, including macrophages, dendritic cells, B cells, and T cells [18]. A previous study has shown that through increasing the PD-L1 expression of macrophages, schistosome worms induce T cells anergy [19].

Breg cells exhibit immunosuppressive function *via* diverse regulatory mechanisms. In addition to schistosome-induced splenic B cells, B-1a cells from the peritoneal cavity (PerC) also have the regulatory capability to induce Treg cells [20]. Moreover, it has been suggested that B cells are essential for Th2 response during infection with *S. mansoni* [21]. In contrast, several studies have indicated that B cells from schistosome-infected mice can inhibit ovalbumin-specific Th2 responses in a partially IL-10-dependent way [8, 22]. Furthermore, in the course of secondary type 2 response to stimulation with soluble egg antigens (SEA), expansion of Tfh cell population is dependent on B cells, since the diminished expansion and activation of Tfh cells are found in the mice administrated with anti-CD20 antibody [23]; whereas other studies have revealed that PD-L^hi^ B cells are critical regulators in Tfh cell programing [14, 24, 25]. All of the above suggest that B cells play a pleiotropic role in regulating immunity to schistosome infection. However, the role of B cells from *S. japonicum-*infected mice in the regulation of CD4^+^ T cell activity has not yet been completely defined.

In order to better understand the characteristics of B cells at different stages of *S. japonicum* infection, we conducted this study and found that the percentages of B-1a and MZB cells decreased and the expression of PD-L1, IL-10, TGF-β and IFN-γ in splenic B cells was upregulated during acute and/or chronic infection. B cells in mice with acute infection had the capability to affect cytokine responses of CD4^+^ T cells, and blocking PD-L1 on B cells from infected mice resulted in a recovery of IL-4-producing CD4^+^ T cells. Moreover, to clarify the role of SEA in inducing regulatory phenotypes of B cells, we performed SEA stimulation *in vitro* and *in vivo* and found that SEA of *S. japonicum* induced higher levels of CD5 in B cells from the spleen but not in PerC B cells. SEA-stimulated splenic and PerC B cells preferentially expressed PD-L1 and TGF-β. Overall, *S. japonicum*-induced B cells exhibit multiple regulatory phenotypes, and they are capable of altering the CD4^+^ T cell response.

## Methods

### Mice and infection

Eight-week-old female BALB/c mice were purchased from the Hubei Provincial Center for Disease Control and Prevention (Wuhan, China). Mice were maintained in a specific pathogen-free condition, and percutaneously infected with 16 ± 2 cercariae of *S. japonicum* shed from infected *Oncomelania hupensis* snails acquired from Nanjing Institute of Schistosomiasis Prevention and Control (Nanjing, China). Mice were sacrificed by CO_2_ asphyxiation at the indicated time for further studies.

### Preparation of soluble antigens

Adult *S. japonicum* were obtained by portal perfusion of mice infected for 8 weeks. The male and female worms were separated under a stereomicroscope. Soluble worm antigen (SWA) was prepared by homogenizing male worms in phosphate-buffered saline (PBS), as previously described [26]. Eggs of *S. japonicum* were isolated from livers of infected mice, placed in PBS and sonicated as previously described for harvesting the soluble fraction used as soluble egg antigens (SEA) [27]. The concentration of antigens was determined by bicinchoninic acid (BCA) assay.

### Cell isolation

Peritoneal cavity cells were obtained by an intraperitoneal injection of 8 ml PBS supplemented with 2% fetal calf serum and 2 ml air, and then passed through a 27 G needle. Single cell suspensions from the spleen were prepared by dispersion through a 70 μm cell strainer (BD Biosciences, San Jose, CA, USA), and erythrocytes depleted by lysis with 0.87% ammonium chloride solution. Total cell numbers were recorded, and cell viability was determined by trypan blue. Splenic B cells and CD4^+^ T cells were isolated using the Mouse Pan-B Cell Isolation Kit (Miltenyi Biotec, Auburn, CA, USA) and the Mouse CD4 T Lymphocyte Enrichment Set (BD Biosciences) following the manufacturer’s instructions, respectively. Briefly, pan B cells were obtained by negative selection (purity 90–94%) using magnetic beads against CD4, CD11c, CD49b, CD90.2, Gr-1 and Ter119. CD4^+^ T cells were enriched by negative selection (purity 92–96%) using magnetic beads against CD8, CD11b, CD45R, CD49b and Ter119.

### Stimulation of murine B cells with soluble antigens *in vitro*

Mouse splenic B cells and PerC washout cells (1.5 × 10^6^ cells per ml) were cultured in medium (RPMI 1640 glutamax; Gibco, Grand Island, NY) containing 5% fetal bovine serum (FBS; Gibco, Grand Island, NY), 5 × 10^−5^ M 2-mercaptoethanol (Sigma-Aldrich, St. Louis, MO, USA) and antibiotics (100 U/ml penicillin and 100 μg/ml streptomycin; Sigma-Aldrich). For investigating the reaction of B cells to SEA *in vitro*, these cells were stimulated with SEA (20 μg/ml, 20 μl SEA in 1 ml culture system), lipopolysaccharide (LPS, 10 μg/ml) or PBS as a control. After cultivation at 37 °C for 24 h, the cells were employed for surface molecules detection.

### Soluble antigens treatment *in vivo*

Mice were intraperitoneally injected with two doses of 100 μg SEA, 100 μg SWA or PBS every seven days. At day 14 after the first injection, peritoneal cavity washout cells and single-cell suspensions from the spleen were harvested for surface staining.

### Antibody blocking and co-culture *in vitro*

Antibody blocking was performed similarly to the method described by Robert et al. [28]. Briefly, isolated splenic B cells (1 × 10^6^ cells per ml) were pre-treated with 10 µg/ml anti-PD-L1 antibody (10F.9G2; Bioxcell, West Lebanon, NH, USA), or isotype antibody (LTF-2; Bioxcell) for 4 h at 37 °C, washed, and then co-cultured with enriched isogenic CD4^+^ T cells at a 1:1 ratio in the presence of rIL-2 (20 ng/ml) (Novus Biologicals, Littleton, CO, USA) for another 24 h. Expression of intracellular cytokines and transcription factors in CD4^+^ T cells was determined as described above.

### Antibody treatment *in vivo*

To block PD-L1 *in vivo*, 100 μg of anti-PD-L1 antibody (10F.9G2; Bioxcell), 100 μg of rat IgG2a isotype control (LTF-2; Bioxcell), or PBS was intraperitoneally injected twice a week, starting at 29 days post-infection (dpi). At 56 dpi, all the mice were sacrificed. Mice were antibody-treated for 4 weeks in total. Splenic cells were prepared for flow cytometry (FCM) analysis, and livers were isolated for pathological examination. Livers were digested at 37 °C with 10% KOH for 20 min and the infection intensity was analyzed. Serum samples were isolated and stored at – 80 °C for cytokine determination.

### Flow cytometry

Single cell suspension was prepared and washed in staining buffer (PBS with 0.02% sodium azide and 2% FBS). Fc receptors were blocked by incubating with anti-CD16/32 antibody (2.4G2; BD Biosciences) for 15 min. For surface marker analysis, cells were incubated with the following monoclonal antibodies: CD19-FITC/APC-Cy7 (1D3); CD5-PE-Cy5 (53–7.3); TGF-β1-PE (TW7-16B4); CD4-FITC/APC/APC-Cy7 (RM4-5); CD62L-APC-Cy7 (MEL-14); CD44-PE (IM7); PD-L1-PE (MIH5) (BD Biosciences); CD21-APC (7E9); and CD23-FITC (B3B4) (Biolegend, San Diego, CA, USA).

For all intracellular cytokine staining, cells were stimulated with 1 μg/ml ionomycin (Sigma-Aldrich), 100 ng/ml phorbol 12-myristate 13-acetate (PMA; Sigma-Aldrich) and 10 μg/ml Brefeldin A (BD Biosciences) for 4 h at 37 °C. Cells were then washed, stained extracellularly, fixed and permeabilized with the intracellular cytokine detection kit (BD Biosciences) according to the manufacturer’s instructions, and then stained with IL-10-PE/APC/BV421 (JES5-16E3), IL-4-PE/APC (11B11) and IFN-γ-PE/PF-Cy7 (XMG1.2) (BD Biosciences).

For transcription factor staining, cells were fixed and permeabilized with the transcription factor staining kit (eBioscience, San Diego, CA, USA) according to the manufacturer’s instructions, and then stained with Foxp3-APC/PE (FJK-16s), T-bet-eFlour660 (4B10), Gata3-PE-Cy7 (TWAJ) and Bcl6-PE (K112-91) (eBioscience).

Dead cells were excluded by staining with the fixable viability dye eFluor 506 (eBioscience). All antibodies were used at an optimal concentration after titration. Gating of cells was based on the specific isotype control as well as fluorochrome minus one (FMO) setting when needed. Flow cytometry was performed using FACSVerse or LSR II (BD Biosciences). Data were analyzed with FlowJo software (BD Biosciences).

### Cytokine measurements

The concentration of cytokines in serum from mice was measured by the BD Biosciences mouse cytokine bead array (CBA) kit as per the manufacturer’s instructions. Briefly, 50 μl of each sample was incubated with mixed capture beads and mouse PE detection reagent for 2 h at room temperature. One ml wash buffer was added into the sample tube followed by centrifugation at 200×*g* for 5 min. The bead pellet was then resuspended with 300 μl wash buffer for FCM analysis. The cytokine concentration in each sample was calculated from the fitting curve with a dilution factor applied.

### Liver pathology

The right lobe of liver from each mouse was fixed in 4% paraformaldehyde and then embedded in paraffin. Five µm sections were dewaxed and stained with Masson’s trichrome. The Masson’s trichrome stain identified the collagen fibers as light blue. Six random (magnification of 100×) digital images were captured from each sample using the Mshot Image Analysis System (Guangzhou Micro-shot Technology Co., Ltd, Guangzhou, China), and the ratio of the collagen fiber area to the total captured area of each section was quantified using ImageJ software in a blinded fashion. Representative images were acquired at a magnification of 200×.

### Real-time PCR

Total RNA was extracted from liver tissues with Trizol reagent (Invitrogen, Carlsbad, CA, USA). cDNA was synthesized using a reverse transcription kit (TOYOB, Tokyo, Japan) according to the manufacturer’s instructions. Real-time PCR was performed using SYBR green master mix (TOYOB) on a MyiQTM2 thermal cycler (Bio-Rad, Hercules, CA, USA). The housekeeping gene glyceraldehyde-3-phosphate dehydrogenase (GAPDH) was used to normalize the real-time PCR data, and the primers used for quantitative PCR were as follows: GAPDH (5ʹ-GTG TTT CCT CGT CCC GTA G-3ʹ and 5ʹ-ATG GCA ACA ATC TCC ACT TT-3ʹ); a-SMA/Acta2 (5ʹ-GAG CGT GAG ATT GTC CG-3ʹ and 5ʹ-GCT GTT ATA GGT GGT TTC G-3ʹ); and Col1a1 (5ʹ-ACA TGT TCA GCT TTG TGG ACC-3ʹ and 5ʹ- TAG GCC ATT GTG TAT GCA GC-3ʹ).

### Statistical analysis

All data are presented as the mean ± standard error (SE) of the mean. A horizontal line with a symbol representing the *P*-value indicates a statistical comparison. We used two-sided Student’s unpaired t-test or Mann–Whitney U-test for comparison of two groups, and Kruskal–Wallis test with Dunn’s *post-hoc* test or one-way ANOVA with Bonferroni’s *post-hoc* test for multiple comparisons, depending on the data distribution (Shapiro–Wilk’s test) and homogeneity of variance (Levene’s test). Specific statistical tests are described in the figure legends. A *P*-value of < 0.05 was considered significant. All statistical analyses were performed using SPSS software (SPSS, Chicago, Illinois, USA).

## Results

### *Schistosoma japonicum* infection modulates B cell differentiation

**Fig. 1:**
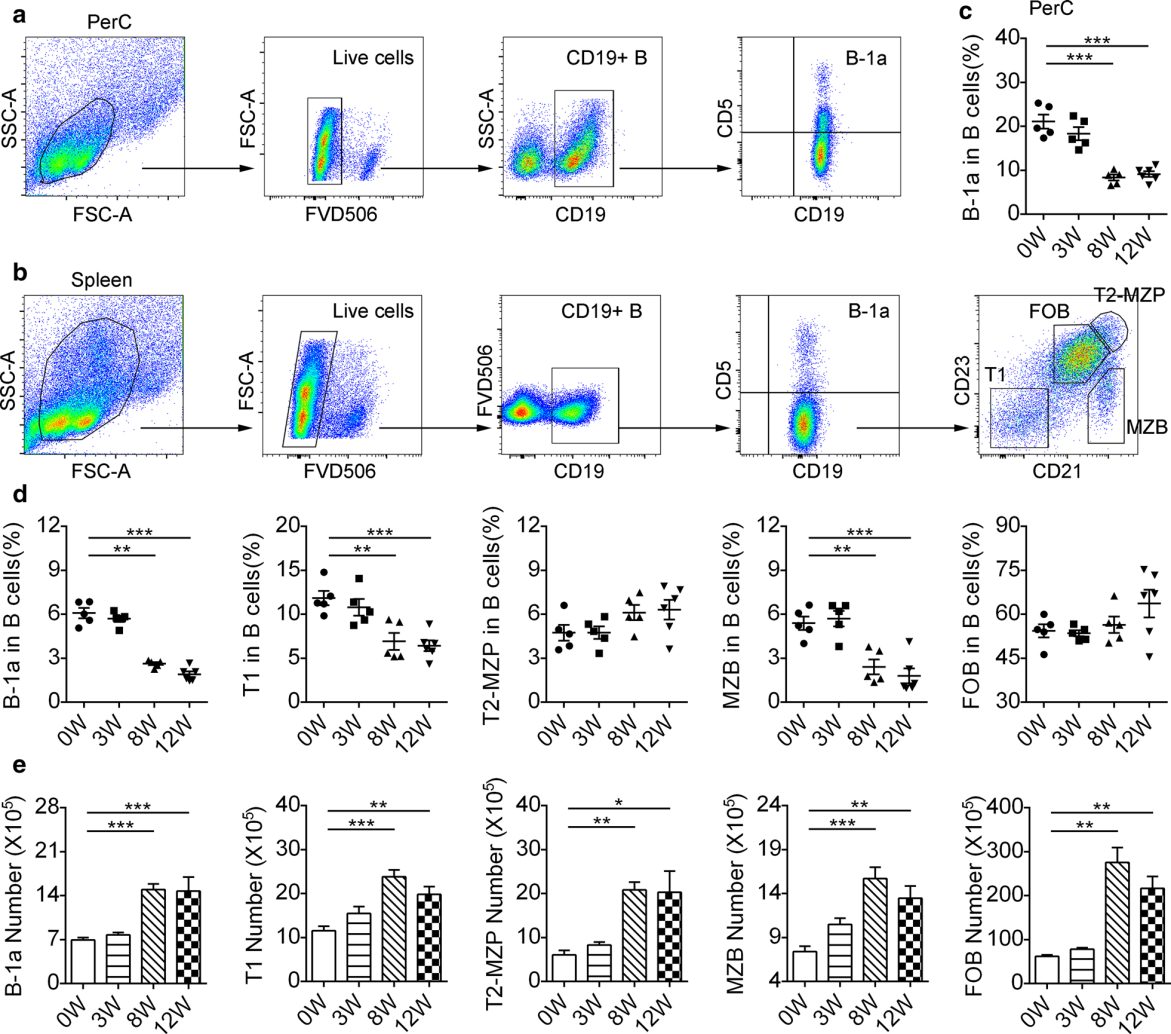
Infection with *Schistosoma japonicum* alters splenic and PerC B-cell composition. Splenic and PerC lymphocytes were isolated from mice at zero (uninfected mice, 0W), three (3W), eight (8W), and twelve weeks (12W) post-infection. Gating schemes for PerC B-1a cells (**a**) and splenic B-cell subsets (**b**) within the CD19^+^ gated B-cell population are shown. Splenic B-cell subpopulations (**b**) are shown: B-1a cells (CD19^+^CD5^+^), T1 B cells (CD19^+^CD5^−^CD21^−^CD23^−^), T2-MZP cells (CD19^+^CD5^−^CD21^+^CD23^+^), MZB cells (CD19^+^CD5^−^CD23^−^CD21^+^) and FOB cells (CD19^+^CD5^−^CD23^+^CD21^−^). The percentages of PerC B-1a cells (**c**) were measured. The percentages (**d**) and the absolute numbers (**e**) of splenic B cell subsets were measured from at least five mice of each time point after infection. All data are representative of at least two independent experiments. *P < 0.05, **P < 0.01, ***P < 0.001

We first investigated the B cell subsets in the PerC and spleen that have been confirmed to play a regulatory role in mice, such as B-1a cells, MZB cells and T2-MZP cells, at different time points after *S. japonicum* infection (Fig. 1a, b). Flow cytometric analysis showed that, at three weeks post-infection (early stage) representing a time point prior to oviposition, the proportions of PerC B-1a cells (Fig. 1c) and the indicated splenic B cell subsets (Fig. 1d) were not significantly different from those in uninfected mice. Likewise, there were no changes in the percentages of T2-MZP cells (*F*_(3, 17)_ = 2.323, *P* = 0.1115) and follicular B (FOB) cells (*F*_(3, 17)_ = 2.15, *P* = 0.1315) at eight (acute stage) or twelve weeks (chronic stage) of infection (Fig. 1d). Besides, the proportions of T1 B cells (*F*_(3, 17)_ = 10.94, *P* < 0.001) and MZB cells (*F*_(3, 17)_ = 16.2, *P* < 0.001) in the infected mice at acute and chronic stage were significantly lower than those of uninfected mice (Fig. 1d). Similarly, the proportions of PerC B-1a cells (*F*_(3, 17)_ = 31.22, *P* < 0.001) (Fig. 1c) and splenic B-1a cells (*F*_(3, 17)_ = 86.9, *P* < 0.001) (Fig. 1d) decreased at acute and chronic stage. The numbers of splenic B cell subsets increased gradually when the infection became acute (Fig. 1e).

### Characterization of splenic and PerC B cells during *S. japonicum* infection

**Fig. 2:**
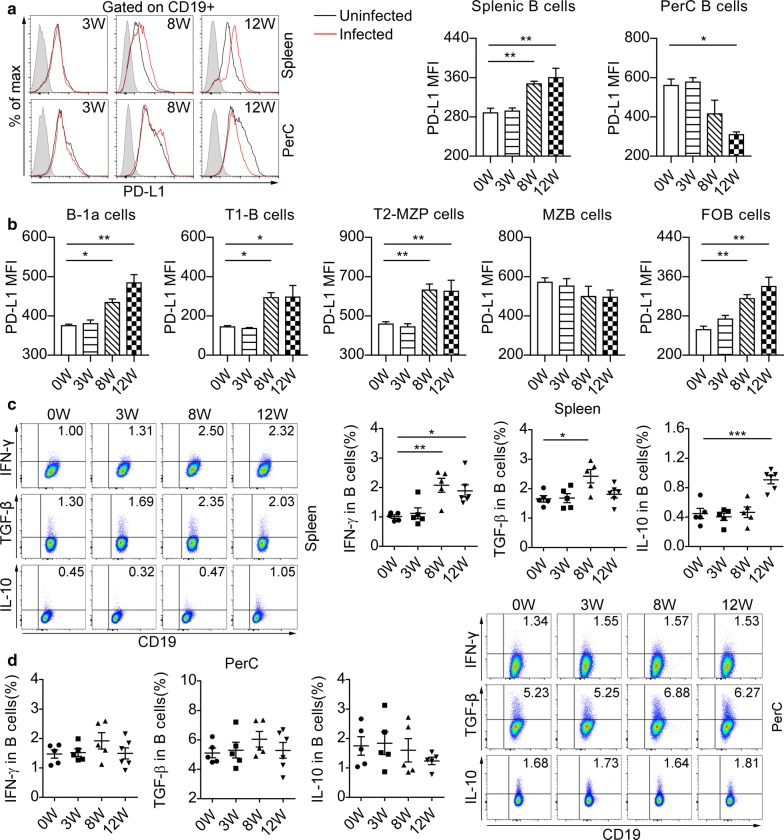
Infection with *Schistosoma japonicum* elevates PD-L1 expression and modulates cytokines profile of splenic B cells. **a** Flow cytometric histograms represent cell surface expression of PD-L1 in splenic and PerC B cells from uninfected mice (black line) and infected mice (red line) at each time point. The shaded histograms represent the FMO control. Significance levels from statistical analysis of mean fluorescence intensity (MFI) of PD-L1 in splenic and PerC B cells are indicated. **b** The MFI of PD-L1 in splenic B cell subsets are summarized. The splenic (**c**) and PerC (**d**) lymphocytes were stimulated with PMA and ionomycin in the presence of Brefeldin A for 4 h and followed by extracellular and intracellular staining. The percentages of IFN-γ^+^, TGF-β^+^ or IL-10^+^ cells gated on CD19^+^ B cells are shown. All data are representative results of two independent experiments with at least 5 mice per group. *P < 0.05, **P < 0.01, ***P < 0.001

To explore the potential of B cells to induce hyporesponsiveness during infection with *S. japonicum*, we investigated B cells from the spleen and peritoneal cavity for immune-regulatory markers. The expression of PD-L1 in splenic B cells was increased at eight and twelve weeks after infection, compared to those from uninfected mice (*F*_(3, 16)_ = 10.6, *P* < 0.001) (Fig. 2a). Interestingly, the expression of PD-L1 in PerC CD19^+^ B cells was similar between uninfected mice and mice infected for either three or eight weeks, but was downregulated in mice at twelve weeks post-infection (*F*_(3, 16)_ = 9.83, *P* < 0.01) (Fig. 2a). We further analyzed the expression of PD-L1 in splenic B cell subsets (Fig. 2b). We did not observe the change of PD-L1 expression in MZB cells (*F*_(3, 16)_ = 0.976, *P* = 0.4285) during *S. japonicum* infection, but acute and chronic infection enhanced the expression of PD-L1 in B-1a cells (*F*_(3, 16)_ = 16.6, *P* < 0.001), T1 B cells (*F*_(3, 16)_ = 7.78, *P* < 0.01), T2-MZP B cells (*F*_(3, 16)_ = 9.5, *P* < 0.001) and FOB cells (*F*_(3, 16)_ = 12.1, *P* < 0.001).

Next, we examined the cytokine production of B cells after *S. japonicum* infection. The percentage of splenic B cells expressing IL-10 was increased at twelve weeks post-infection (*F*_(3, 17)_ = 15.84, *P* < 0.001), while TGF-β in splenic CD19^+^ B cells was elevated at eight weeks after infection but declined thereafter (*F*_(3, 17)_ = 4.979, *P* < 0.01) (Fig. 2c). Schistosome infection induced elevated percentages of IFN-γ-expressing splenic B cells at both acute and chronic stages (*F*_(3, 17)_ = 7.954, *P* < 0.001) (Fig. 2c). Moreover, there were no differences in IL-10, TGF-β, or IFN-γ expression of PerC CD19^+^ B cells between infected and uninfected mice (Fig. 2d).

### SEA-stimulated B cells acquire a regulatory phenotype

**Fig. 3:**
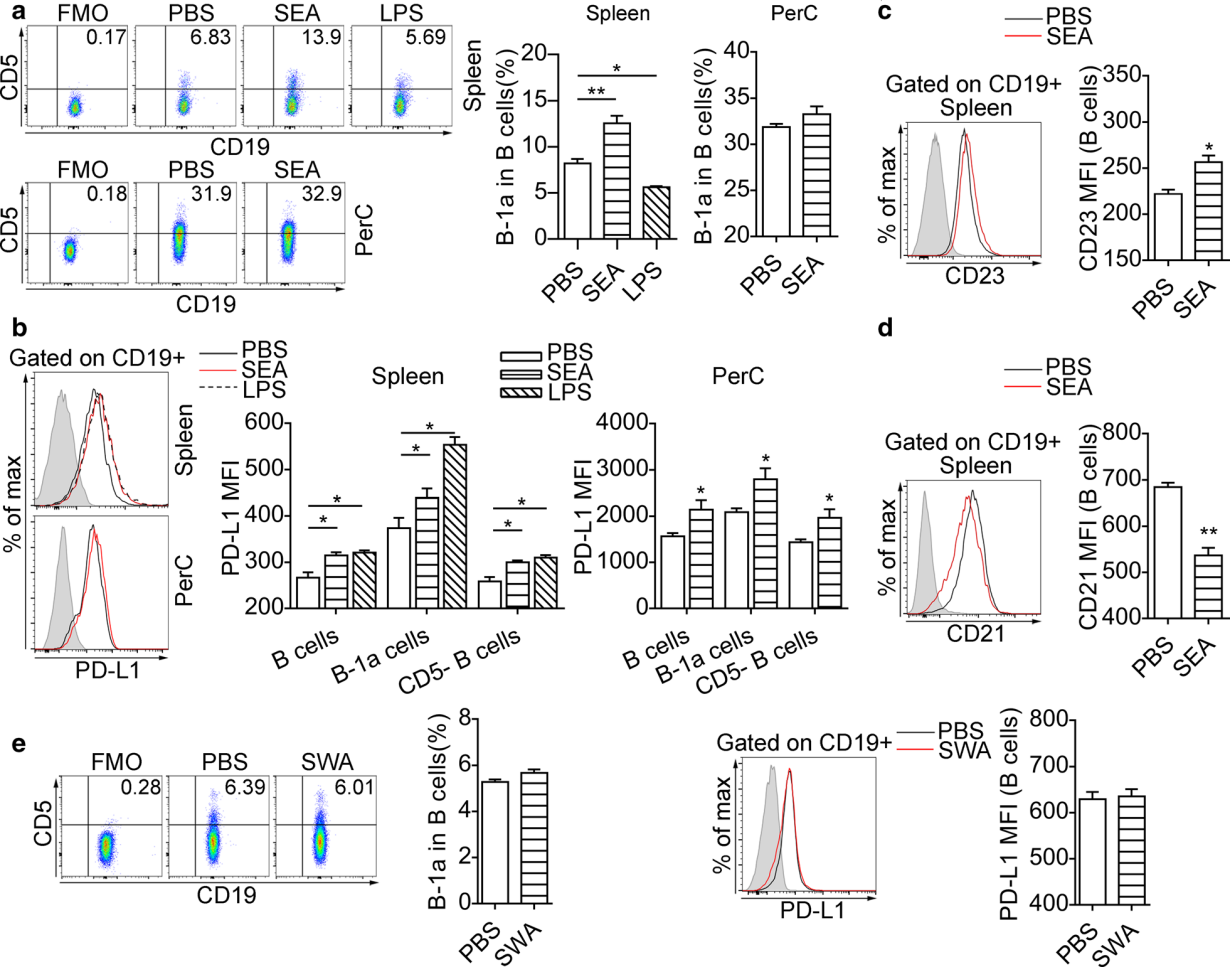
SEA drives CD5 and PD-L1 expression of B cells *in vitro*. Splenic B cells and PerC washout cells from uninfected mice were cultured for 24 h in the presence of PBS or indicated stimuli. **a** Representative dot plots indicate the expression of CD5 on gated CD19^+^ B cells from spleen and peritoneal cavity and significance levels from statistical analyses are indicated. **b** Flow cytometric histograms represent cell surface expression of PD-L1 in splenic and PerC B cells, and significance levels from statistical analyses of PD-L1 MFI of splenic and PerC B-cell are indicated. The expression of CD23 (**c**) and CD21 (**d**) in splenic B cells was measured at the end of culture. The shaded histograms represent the FMO control. In **e** and **f**, splenic B cells were cultured for 24 h in the presence of PBS or SWA (20 μg/ml). **e** Representative dot plots indicate the expression of CD5 on gated CD19^+^ B cells from spleen and significance levels from statistical analyses are indicated. **f** Flow cytometric histograms represent cell surface expression of PD-L1 in splenic B cells, and significance from statistical analyses of PD-L1 MFI of splenic B-cell are indicated. All data are representative results of two independent experiments with at least 4 mice per group. *P < 0.05, **P < 0.01

We next tested whether SEA could induce a regulatory phenotype in B cells. To test the induction of PD-L1 in B cells by SEA *in vitro*, purified B cells from uninfected mice were cultured for 24 h with the stimulation of SEA (20 μg/ml) (designated SEA-B) or LPS (10 μg/ml) (LPS-B), and negative control B cells were cultured with PBS (PBS-B). We found that splenic SEA-B cells expressed higher level of CD5 compared to PBS-B cells (*F*_(2, 9)_ = 41.6, *P* < 0.001), whereas the expression of CD5 was not significantly increased in PerC SEA-B cells (*t*_(6)_ = 1.54, *P* = 0.1882) (Fig. 3a). LPS failed to induce an increase in the percentage of CD5^+^ B cells (Fig. 3a). The splenic and PerC SEA-B cells expressed higher levels of surface PD-L1 (Fig. 3b). The level of CD23 (*t*_(6)_ = 3.96, *P* < 0.05) was increased in splenic SEA-B cells (Fig. 3c), but the expression of CD21 in splenic SEA-B cells (*t*_(6)_ = 5.341, *P* < 0.01) was decreased (Fig. 3d). Besides, SWA failed to affect the expression of CD5 and PD-L1 in splenic B cells *in vitro* (Fig. 3e, f).

**Fig. 4:**
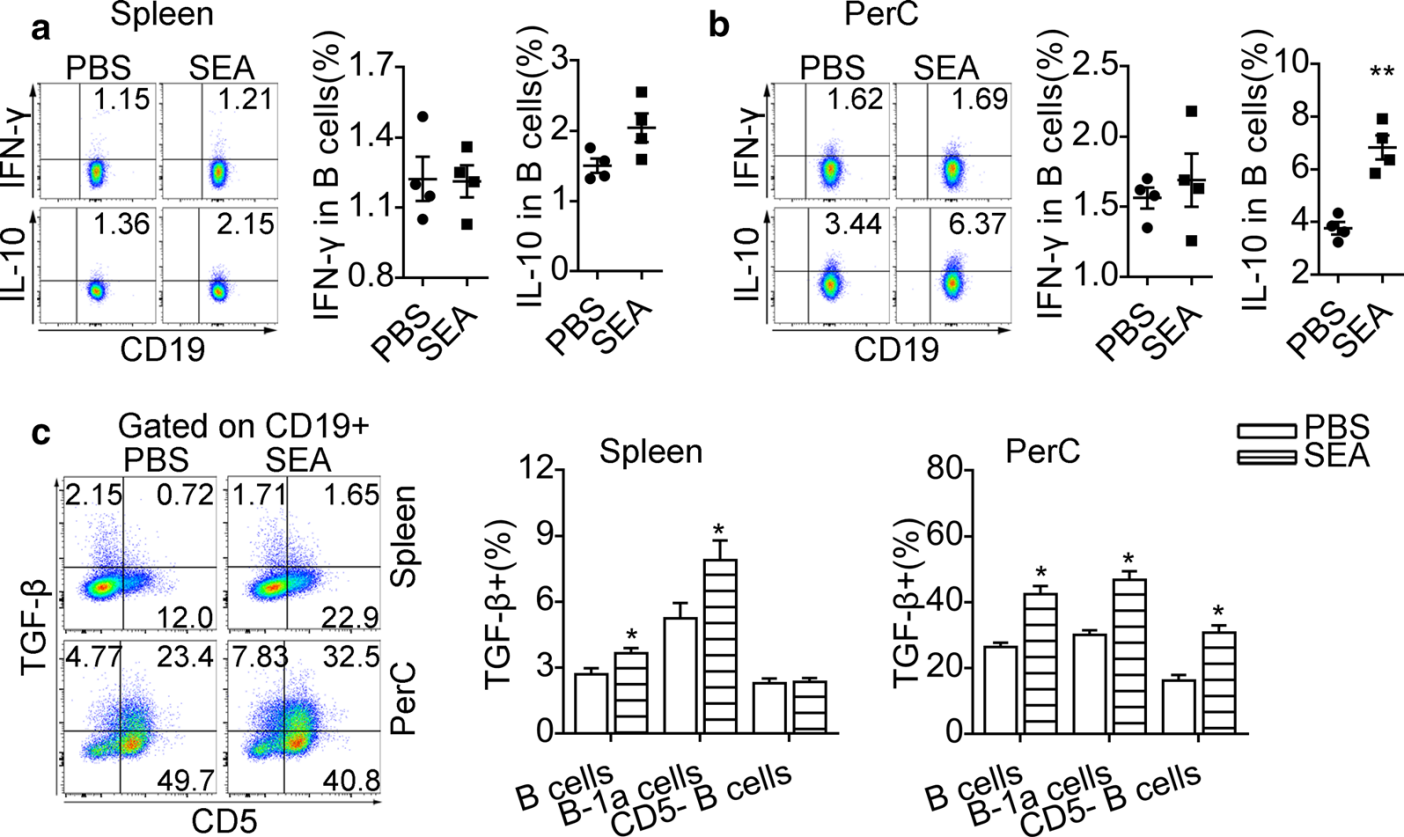
SEA-stimulated B cells acquire elevated expression of IL-10 and TGF-β *in vitro*. Splenic B cells and PerC washout cells from uninfected mice were stimulated with SEA for 24 h. Intracellular IL-10 and IFN-γ staining were performed after the restimulation with PMA and ionomycin in the presence of Brefeldin A. Representative flow cytometric plots and significance from statistical analyses of the percentages of IL-10^+^ B cells and IFN-γ^+^ B cells in splenic B cells (**a**) and PerC B cells (**b**) are shown. **c** Representative plots of CD5 and TGF-β expression on CD19^+^ B cells and the percentages of B cell subsets expressing TGF-β. All data are representative results of two independent experiments with at least 4 mice per group. *P < 0.05, **P < 0.01

Additionally, SEA stimulation for 24 h did not significantly enhance IL-10 expression in splenic B cells (*t*_(6)_ = 2.384, *P* = 0.0545) (Fig. 4a), but drove the expression of IL-10 in PerC B cells (*t*_(6)_ = 5.974, *P* < 0.01) (Fig. 4b). Our data also showed that SEA treatment *in vitro* did not affect the intracellular IFN-γ expression in splenic and PerC B cells (Fig. 4a, b). After the stimulation with SEA, splenic B-1a cells (*t*_(6)_ = 4.603, *P* < 0.05) but not splenic CD5^-^ B cells (*t*_(6)_ = 0.369, *P* = 0.7248) showed significantly increased surface expression of TGF-β (Fig. 4c). Both B-1a cells (*t*_(6)_ = 11.23, *P* < 0.05) and CD5^-^ B cells (*t*_(6)_ = 10.16, *P* < 0.05) in PerC had significantly increased expression of TGF-β after SEA treatment *in vitro* (Fig. 4c).

**Fig. 5:**
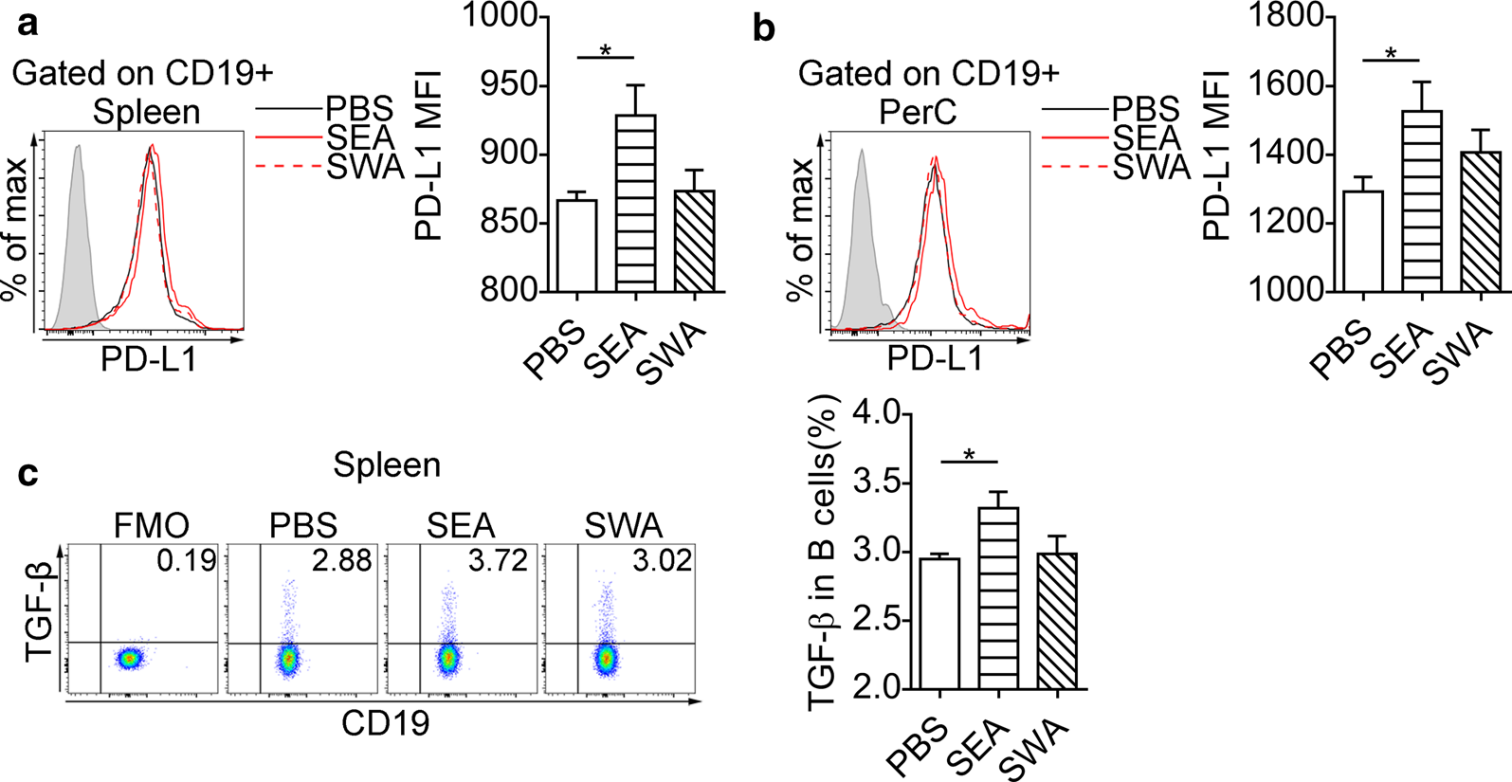
SEA induces expression of PD-L1 and TGF-β on B cells *in vivo*. Flow cytometry histograms represent cell surface expression of PD-L1 in splenic B cells (**a**) and PerC B cells (**b**), and significance levels from statistical analysis of PD-L1 MFI of splenic (**a**) and PerC B cells (**b**) are shown. The shaded histograms represent the FMO control. **c** Representative plots of TGF-β surface expression in CD19^+^ B cells and the percentages of TGF-β expressing B cells. The data are representative results of two independent experiments with at least 4 mice per group. *P < 0.05

We further examined if intraperitoneal injection of SEA was sufficient to induce expression of PD-L1 in B cells. Intraperitoneal injection of SEA into mice increased the expression of PD-L1 in splenic B cells (*F*_(2, 10)_ = 4.18, *P* < 0.05) (Fig. 5a) and PerC B cells (*F*_(2, 10)_ = 4.43, *P* < 0.05) (Fig. 5b), as well as the expression of TGF-β (*F*_(2, 12)_ = 3.98, *P* < 0.05) in splenic B cells (Fig. 5c). In contrast, SWA injection did not affect the expression of PD-L1 in B cells (Fig. 5a, b).

### B cells from *S. japonicum-*infected mice modulate CD4^+^ T cell response

**Fig. 6:**
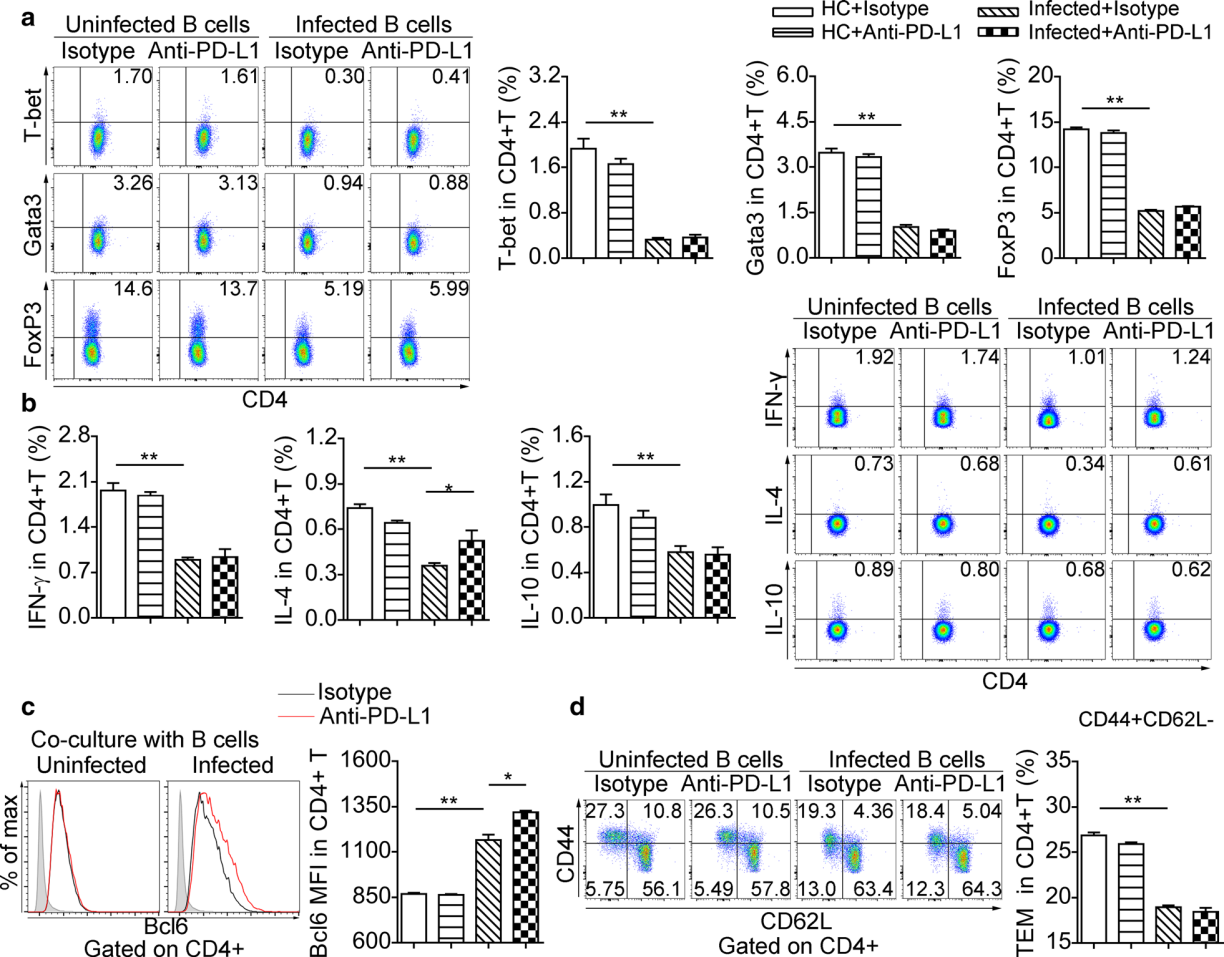
B Cells from infected mice modulate the phenotype of CD4^+^ T Cells *in vivo*. Purified splenic B cells from uninfected mice and infected mice (eight weeks) were treated with isotype antibody or anti-PD-L1 antibody respectively prior to co-culture with CD4^+^ T cells. **a** Representative dot plots of T-bet, Gata3, and FoxP3 in CD4^+^ T cells and significance levels from statistical analysis. **b** Representative flow cytometric plots of IL-10, IL-4 and IFN-γ in CD4^+^ T cells and significance levels from statistical analyses. **c** Representative flow cytometric histograms of Bcl6 expression in CD4^+^ T cells and significance levels from statistical analysis. The shaded histograms represent the FMO control. **d** Representative dot plots of CD44 *versus* CD62L gated on CD4^+^ T cells (CD44^+^CD62L^−^ T effector memory, TEM) and significance levels from statistical analysis. All data are representative results of two independent experiments with at least 4 mice per group. *P < 0.05, **P < 0.01

We performed splenic CD4^+^ T cell and B cell co-cultures to investigate the suppressive functions of B cells from mice at eight weeks post-infection. The frequencies of T-bet^+^ (*F*_(3, 18)_ = 74.8, *P* < 0.01), Gata3^+^ (*F*_(3, 16)_ = 236, *P* < 0.01) and FoxP3^+^ (*F*_(3, 20)_ = 639, *P* < 0.01) in CD4^+^ T cells (Fig. 6a), as well as the percentages of CD4^+^ T cells producing IFN-γ (*F*_(3, 16)_ = 43, *P* < 0.01), IL-4 (*F*_(3, 14)_ = 14.9, *P* < 0.01) and IL-10 (*F*_(3, 16)_ = 37.1, *P* < 0.01) (Fig. 6b), significantly decreased after co-culture with B cells from infected mice. In contrast, B cells from infected mice enhanced the expression of Bcl6 in CD4^+^ T cells (*F*_(3, 18)_ = 240, *P* < 0.01) (Fig. 6c). Additionally, B cells from infected mice were prone to generate fewer CD4^+^ T effector memory (TEM, CD44^+^CD62L^−^) cells than B cells from uninfected mice (*F*_(3, 20)_ = 198, *P* < 0.01) (Fig. 6d).

Based on the elevated expression of PD-L1 in splenic B cells at eight weeks of experimental *S. japonicum* infection (Fig. 2b), we blocked PD-L1 on B cells before co-culture with CD4^+^ T cells. Anti-PD-L1 antibody pre-treatment did not recover the expression of T-bet, Gata3, FoxP3 (Fig. 6a), IFN-γ and IL-10 (Fig. 6b) in CD4^+^ T cells, whereas the expression of IL-4 and Bcl6 was significantly increased in CD4^+^ T cells (Fig. 6b, c). Also, anti-PD-L1 antibody treatment did not affect suppression in CD4^+^ T effector memory cells mediated by B cells from infected mice (Fig. 6d).

### PD-L1 blocking partially expands the Th2 response in *S. japonicum-*infected mice

**Fig. 7:**
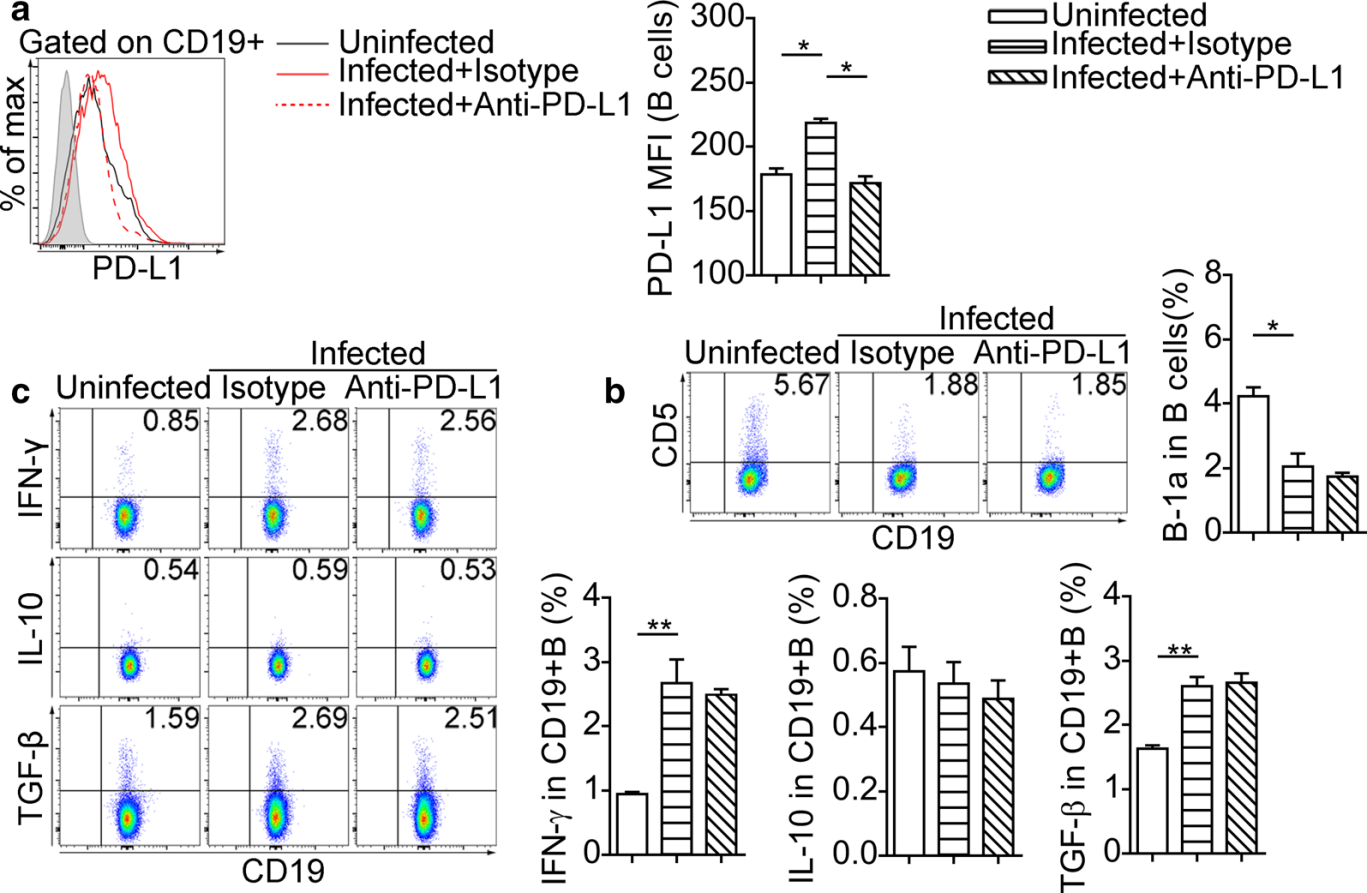
PD-L1 blocking fails to alter the regulatory molecules in splenic B-cell during infection. **a** Overlay of representative histograms show PD-L1 expression in splenic CD19^+^ B cells. Representative dot plots show the expression of CD5 (**b**), IFN-γ, IL-10 and TGF-β (**c**) in splenic CD19^+^ B cells. Bar graphs indicate the results from one of two independent experiments with ≥ 5 mice in each group. *P < 0.05, **P < 0.01

To further understand the role of PD-L1 in regulating host immunity of schistosomiasis, we used anti-PD-L1 antibody in the *S. japonicum* infection model. The PD-L1 MFI of splenic CD19^+^ B cells from infected mice decreased after anti-PD-L1 antibody treatment (*F*_(2, 12)_ = 29.5, *P* < 0.05) (Fig. 7a). Nevertheless, the splenic CD19^+^ B cells from infected mice treated with anti-PD-L1 antibody and isotype antibody were similar in the proportion of B-1a cells (Fig. 7b) and the expression of IFN-γ, IL-10 and TGF-β (Fig. 7c).

**Fig. 8:**
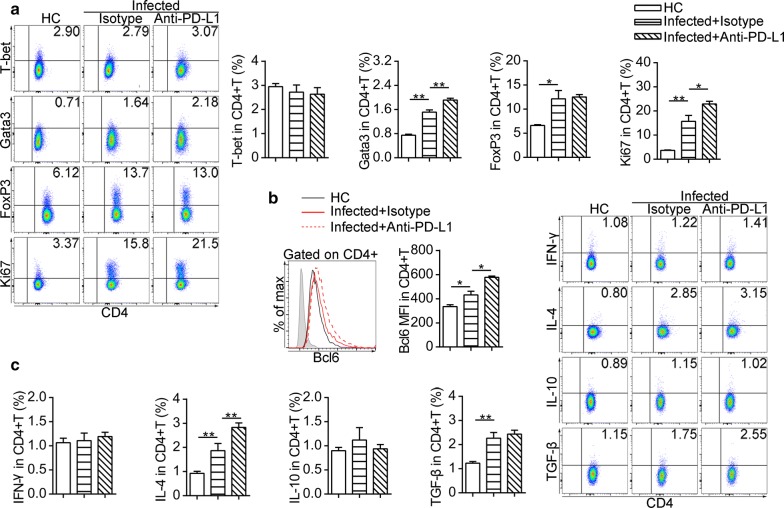
*In vivo* blocking of PD-L1 partially expands Th2 response after *S. japonicum* infection. **a** Flow cytometric dot plots represent expression of T-bet, Gata3, FoxP3 and Ki67 in splenic CD4^+^ T cells. **b** Flow cytometric histograms represent Bcl6 expression in splenic CD4^+^ T cells from uninfected mice (black line) and infected mice treated with isotype antibody (red line) and infected mice treated with anti-PD-L1 antibody (dashed line). The shaded histograms represent the FMO control. **c** Flow cytometric dot plots showing expression of IFN-γ, IL-4, IL-10 and TGF-β in splenic CD4^+^ T cells. All bar graphs indicate results from one of two independent experiments with ≥ 5 mice in each group. Experiments were performed two times with *n* = 10–12 per group. *P < 0.05, **P < 0.01

However, PD-L1 blocking significantly increased the proportion of Gata3^+^CD4^+^ T cells (*F*_(2, 13)_ = 72.1, *P* < 0.01) and the proliferation of CD4^+^ T cells in the spleen (Fig. 8a). Also, with anti-PD-L1 antibody treatment, CD4^+^ T cells from infected mice exhibited a higher level of Bcl6 (*F*_(2, 12)_ = 32.6, *P* < 0.01) (Fig. 8b) and secreted more IL-4 (*F*_(2, 13)_ = 26.3, *P* < 0.01) (Fig. 8c). The expression of T-bet, FoxP3 (Fig. 8a), IFN-γ, IL-10 and TGF-β (Fig. 8c) in CD4^+^ T cells from infected mice with anti-PD-L1 antibody treatment were similar to those found in infected mice treated with the isotype antibody.

**Fig. 9:**
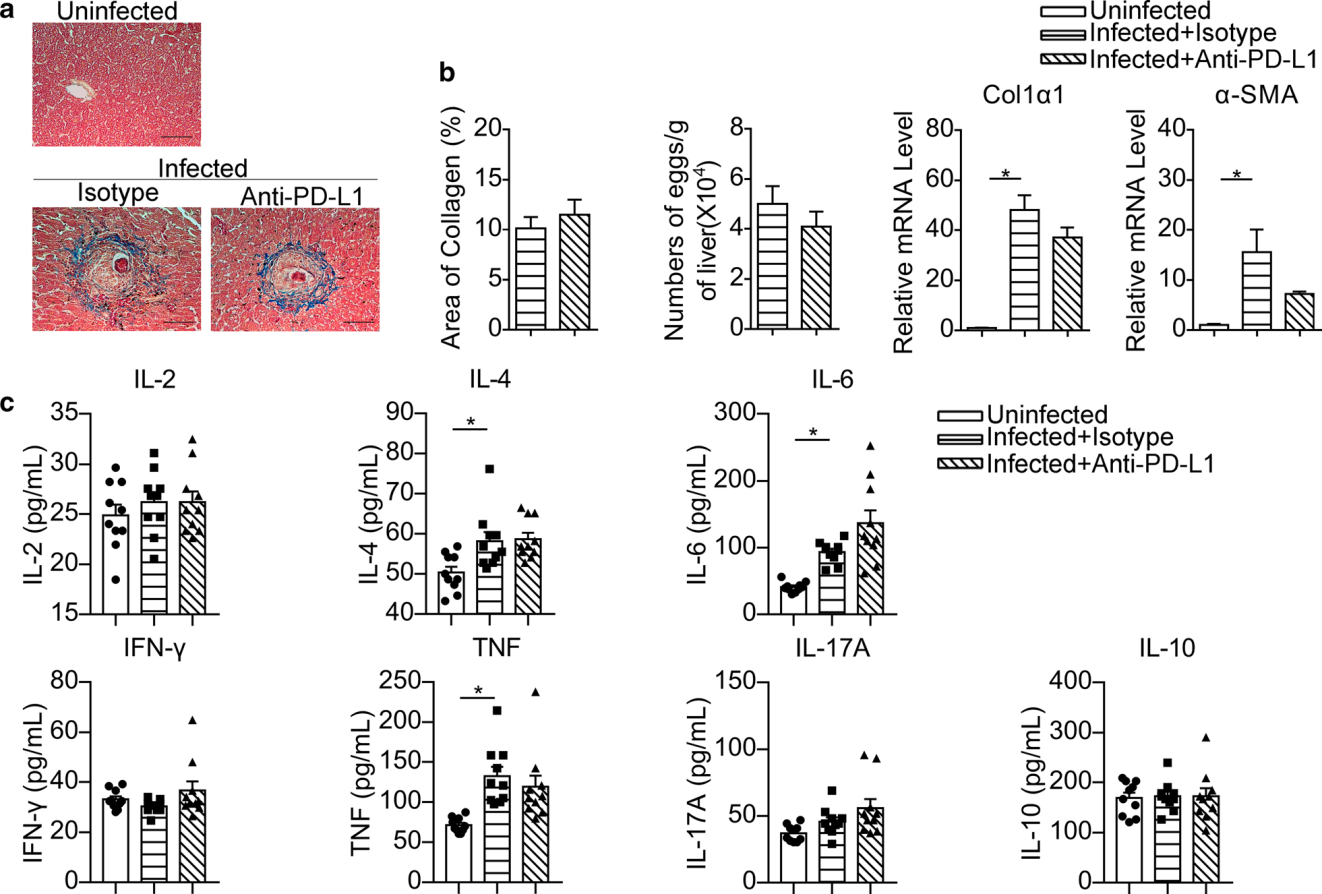
PD-L1 blocking fails to affect hepatic pathology and serum cytokines during infection. **a** Representative images of Masson’s trichrome staining for hepatic fibrosis analysis (original magnification of 200×). **b** Quantification of hepatic collagen deposition and egg burden are shown, and total RNA was extracted from livers and analyzed by RT-PCR for the expression of Col1a1 and a-SMA. In **a** and **b**, the data are representative results of two independent experiments with at least 4 mice per group. **c** The serum levels of IL-2, IL-4, IL-6, IL-10, IL-17A, TNF and IFN-γ were assayed by CBA. The data represent the cumulative results of two independent experiments. *P < 0.05. *Scale-bars*: **a**, 100 μm

In addition, hepatic pathology indicated by collagen and a-SMA expression, as well as hepatic egg burden, were not significantly affected by anti-PD-L1 antibody treatment in schistosome-infected mice (Fig. 9a, b). No significant differences were found in the levels of IL-2, IL-4, IL-6, IL-10, IL-17A, IFN-γ and TNF in serum between infected mice treated with anti-PD-L1 antibody and isotype antibody (Fig. 9c).

## Discussion

It has been well accepted that various helminth infections lead to a strong and comprehensive immune modulation coming down to the whole process of immune response, including almost all the natural and adaptive immunity of their hosts [2]. Enhanced effective CD4^+^ Th cell response has been clearly illustrated in human infection and experimental schistosome models, which is initiated as a Th1 response before abundant oviposition by female worms. At six to eight weeks post-infection, this response switches to Th2, and downmodulation of the Th2 and Th1 response is observed generally at ten weeks, as an essential mediation to favor the survival of both hosts and parasites [1, 29].

Apart from commonly known Treg cells that predominate during the chronic stage of schistosomiasis, regulatory functions of B cells are also demonstrated in the process of schistosomiasis. Previous studies [30, 31] have shown that the deficiency of B cell or B-1a cell has led to a higher mortality in schistosome-infected mice, as well as that B-1a cells regulate the IL-5 and IFN-γ production of Th cells and control the size of ova granulomas during infection. Besides, CD1d^hi^ B cells from *S. mansoni*-infected mice have been shown to function in dampening inflammation in allergic airway inflammation [5]. Murine *S. japonicum* infection has been reported to downregulate allergic airway inflammation through reducing Th17 and Th2 effector cytokines in the lungs [32], but few studies focus on the immune regulatory role of B cells during *S. japonicum* infection. Moreover, Rosser et al. [12] have proposed that the immunosuppression of B cells is not achieved by a specific Breg subset with a particular phenotype, but by the outcome of the interaction between multiple B cell populations and other cells in the immune system, although the Breg subsets have been described as T2-MZP cells [8] and MZB cells [5, 9] in *S. mansoni*-infected mice. Here we found that the proportions of B cell populations with regulatory potential, such as B-1a and MZB cells, were markedly reduced during *S. japonicum* acute and chronic infection. The percentages of B cell subsets with regulatory features were not upregulated in *S. japonicum* infection models, but the numbers of splenic B-1a cells, T2-MZP cells and MZB cells increased progressively when the infection became acute, and these increases were likely due to the development of splenomegaly in schistosome-infected mice [33] and B cell expansion [34].

A previous study has shown that B-1a cells in the peritoneal cavity spontaneously express IL-10, which is further increased in LPS-stimulated PerC B-1a cells [35]. The changes of PerC B-1a cells during early *S. mansoni* infection remain controversial [36–38]. Velupillai et al. [37] have reported that B-1a cell outgrowth depends on the mouse strain during *S. mansoni* infection, and at early and acute infection, the frequencies of PerC B-1a cells change differently among various strains of infected mice. Besides, the percentages of splenic B-1a cells increase in C3H/HeN mice during acute and chronic *S. mansoni* infection [37, 39]. Here, we observed that the proportions of PerC and splenic B-1a cells remained relatively constant at three weeks of *S. japonicum* infection, whereas these proportions were significantly reduced during acute and chronic schistosomiasis. Overall, the outgrowth of B-1a cells in murine schistosomiasis was probably not only related to mouse strain, but also associated with schistosome species. Since B cells are crucial in egg-induced granulomatous response during schistosomiasis [6], our findings suggest that the immunoregulatory mechanism of B cells in *S. japonicum*-infected mice might be different from that in *S. mansoni* infections, although the change of hepatic fibrosis led by B cell-depletion in *S. mansoni-*infected mice was similar to that in mice infected with *S. japonicum* [30].

It has been reported that Breg cells can suppress the immune response by secreting inhibitory cytokines and interacting with surface molecules of the target cells [40]. LPS-stimulated B cells can inhibit Th1 response and prevent autoimmune diabetes by secreting TGF-β [13]. IFN-γ may play diverse roles at different stages of the immune response since it has the capabilities of both inhibiting and stimulating Treg cells [41, 42]. In the present study, IL-10 expression in splenic B cells was enhanced during the chronic stage, which is consistent with a previous report in *S. mansoni* infection models [5]. Expression of TGF-β in splenic B cells was increased in the acute stage of infection but declined after twelve weeks of infection. Schistosome infection also elevated IFN-γ production in splenic B cells in both acute and chronic stages, but there was no change in the expression of IL-10, TGF-β, or IFN-γ in PerC CD19^+^ B cells during schistosome infection, which could be explained by the heterogeneity and distinct composition of B cells derived from the spleen and peritoneal cavity [43]. These B cells responded differentially to parasite infection and a previous report [44] supports this finding by showing that PerC B cells activated by anti-CD40 and LPS secreted more IL-10 and IL-6 compared to splenic B cells. PD-L1 expression in splenic B cells was elevated during acute and chronic infection, whereas the expression of PD-L1 in PerC B cells was decreased during chronic schistosomiasis. The difference in PD-L1 expression of B cells during infection might be due to the higher basic level of PD-L1 in PerC B cells compared to that in splenic B cells [45] and the infection-induced migration of PerC B-1a cells to the liver [46].

Moreover, previous studies have confirmed that SEA can directly interact with B cells to enhance their regulatory potential shown as increasing IL-10 expression [9, 47]. CD23 is the IgE Fc receptor which can regulate allergen-induced airway inflammation [48], and splenic B cells can be subdivided into T2-MZP cells and MZB cells based on the expression of CD21 and CD23. CD23 expression levels of Breg cells are diverse in different disease models [22]. In our study, we found that *S. japonicum* SEA stimulation enhanced the expression of IL-10, TGF-β, CD5, CD23 and PD-L1 in PerC B cells or splenic B cells, which have been identified as the markers of Breg cells. Although SEA had the capability of increasing CD5 and IL-10 expression in PerC B cells *in vitro*, a reduced proportion of PerC B-1a cells and a slightly decreased IL-10 expression in PerC B cells were observed during schistosome infection in our study. This differential effect on the expression of CD5 and IL-10 in PerC B cells resulting from SEA stimulation *in vitro* and infection *in vivo* might be due to the fact that PerC B-1a cells identified by surface marker CD5 were competent to secrete a large amount of IL-10 [35], and that PerC B-1a cells migrated into the liver to improve hepatic fibrosis induced by schistosome infection [46]. Beyond that, the findings from *in vivo* and *in vitro* experiments have suggested that the dynamic changes of these cytokines and surface molecules in B cells during schistosome infection might be influenced not only by the stimulation of SEA but also the internal dynamics of the immune system disturbed by infection [49].

We also found the suppression of Th1 and Th2 cells by splenic B cells from acutely infected mice in co-cultures with CD4^+^ T cells. A previous study has reported that *S. japonicum* infection promotes the expansion of Tfh cells [50]. The interactions between B cells and naïve T cells are essential for the generation of Tfh cells [51, 52]. Nevertheless, several studies have reported that PD-L1-expressing B cells regulate Tfh cells [14, 24, 25]. Herein, we demonstrated that blocking PD-L1 on B cells from acutely infected mice enhanced the expansion of Bcl6 that is described as being the critical transcription factor for Tfh cell differentiation [53]. Additionally, anti-PD-L1 treatment of splenic B cells from acutely infected mice before co-culture partially restored the expression of IL-4 in T cells, whereas Th2 transcription factor Gata3 expression was unchanged. These might be related to the fact that Tfh cells are the primary IL-4-producing cells during helminth infection [54, 55].

Although PD1 signaling in B cells has been demonstrated to inhibit the proliferation and cytokine production of activated B cells [56], we did not find the effects of systematic PD-L1 blocking on the proportion of B-1a cells and cytokine production in splenic B cells during *S. japonicum* infection. PD-L1 blocking in mice after infection only reduced PD-L1 MFI of splenic B cells from infected mice, and this might be because treatment with anti-PD-L1 antibody *in vivo* affected the binding of fluorescent antibodies to PD-L1. Studies in mice with schistosome and *Litomosoides sigmodontis* infection have indicated that Th2 hypo-responsiveness is not related to PD-1 and PD-L1 interaction [22, 57, 58]. However, other studies have suggested that PD-1 is necessary for the Th2 hyporesponsiveness during schistosome infection [19, 59]. Our findings from the PD-L1 blocking experiment *in vivo* were consistent with the latter, as we observed that PD-L1 blocking not only enhanced the expression of IL-4 and Bcl6 in splenic CD4^+^ T cells but also increased the expression of Th2 transcription factor Gata3. These were caused not just by blocking PD-L1 on B cells, since a previous study reported that *S. mansoni* worms selectively increased the expression of PD-L1 in macrophages [19]. Deficiency of PD-L1 specific in B cells would be useful to clarify the role of PD-L1 of B cells during schistosome infection.

The intervention of anti-PD-L1 antibody after oviposition did not exert an influence on hepatic egg burden, and there was no appreciable pathological change indicated by hepatic collagen deposition. It is well known that various resident or recruited inflammatory cells are responsible for the activation of hepatic stellate cells and contribute to liver fibrosis [60]. Our data might imply that only the intervention of PD-L1 could not impact on the pathogen-associated fibrosis in the liver, although the CD4^+^ T cell response was modulated by PD-L1 blockade.

## Conclusions

Our study revealed that different schistosome species might result in the distinct differentiation of B cell subsets. *Schistosoma japonicum* infection could induce the regulatory function of B cells that interact with T cells in an intricate way and display multiple regulatory phenotypes. Further investigation of the role of PD-L1 specific in B cells in modulating T cell activation in schistosome infection should be helpful for the control of other T cell-mediated diseases.

## Data Availability

All data generated or analyzed during this study are included in this published article.

## Abbreviations

Breg: regulatory B cells
CBA: cytokine bead array
dpi: days post-infection
FBS: fetal Bovine Serum
FCM: Flow cytometry
FMO: fluorochrome minus one
FOB: follicular B cells
LPS: lipopolysaccharide
MFI: mean fluorescence intensity
MZB: marginal zone B cells
PerC: peritoneal cavity
PMA: phorbol 12-myristate 13-acetate
SE: standard error
SEA: soluble egg antigens
SWA: soluble worm antigen
T2-MZP: transitional 2 marginal zone B precursor cells
TEM: T effector memory cells
Treg: regulatory T cells
